# The Factors Associated With Readmission in One Year Among Patients With Chronic Heart Failure: A Retrospective Cohort Study

**DOI:** 10.7759/cureus.109465

**Published:** 2026-05-22

**Authors:** Zheng Feng, Hui Mengyuan, Cheng Lin, Liu Lisha, Liu Qiao, Lu Jing, Liao Liqin

**Affiliations:** 1 Department of Cardiovascular Medicine, Nursing Department, The Third Xiangya Hospital, Central South University, Changsha, CHN; 2 Department of Cardiovascular Medicine, Northern Jiangsu People's Hospital, Yangzhou, CHN; 3 School of Graduate Studies, Xiangya School of Nursing, Central South University, Changsha, CHN; 4 Department of Cardiovascular Medicine, Huayuan People's Hospital, Huayuan, CHN

**Keywords:** chronic heart failure, disease duration, ef: ejection fraction, medication literacy, rate of readmission

## Abstract

Objective

This study aimed to identify factors associated with readmission in one year among patients with chronic heart failure (CHF), focusing on the association between medication literacy and readmission.

Methods

This retrospective cohort study included CHF patients diagnosed and treated at three tertiary hospitals in Hunan, China. The Chi-squared test or Fisher's exact test was used for univariate analysis. Binary logistic regression was applied to identify the factors influencing all-cause readmission within one year.

Results

A total of 480 CHF patients were included. Of note, 58.1% of them (279 CHF patients) were readmitted to the hospital for any cause within one year. The all-cause readmission rates at one month, three months, six months, and one year were 19.7%, 35.8%, 46.9%, and 58.1%, respectively, while the corresponding proportions for HF readmissions were 10.8%, 20.7%, 28.4%, and 37.6%. Medication literacy was an independent risk factor for one year all-cause readmission (odds ratio (OR)=0.411, 95% confidence interval (CI)=0.134-0.743,* p*=0.013 ), and the following factors were associated with increased risk of all-cause readmissions within one year: disease duration ≥10 years (OR=5.961, 95% CI=1.981-17.939, *p*=0.001), myocardial infarction (OR=2.818, 95% CI=1.486-5.344, *p*=0.002), New York Heart Association Functional Classifications (NYHA class) III (OR=2.794, 95% CI=1.442-5.411, *p*=0.002), NYHA class IV (OR=4.112, 95% CI=1.952-8.664, *p<*0.001), and heart failure with mid-range ejection fraction (HFmrEF) (OR=2.463, 95% CI=1.241-4.889, *p*=0.010).

Conclusions

Our study found that, in addition to longer disease duration, myocardial infarction, higher NYHA class, and HFmrEF, lower medication literacy may be associated with an increased risk of all-cause readmission in patients with CHF. This new finding provides guidance for the future development of prevention and intervention programs aimed at improving the well-being of CHF patients.

## Introduction

Heart failure (HF) is a serious global health concern associated with high morbidity and mortality, affecting approximately 64.3 million people worldwide [1] and 8.9 million people in China [2]. The prevalence and incidence of chronic heart failure (CHF) are expected to increase annually due to rapid economic development, lifestyle changes, and medical advances that have prolonged the survival of CHF patients [3]. CHF is a leading cause of hospitalization, representing a substantial burden on the healthcare system [4]. It is also the most common reason for readmission in both patients with an initial CHF diagnosis [5] and those hospitalized for other conditions [6]. Specifically, various studies have reported that the one-year readmission rate for CHF is high [7,8].

Given the high prevalence and disease burden of CHF readmission, it is important to understand the factors associated with hospital readmission within one year for CHF patients to guide targeted interventions aimed at improving patient well-being and reducing the disease burden. A review of previous studies on factors influencing one-year CHF readmission has identified numerous variables, including gender [9], high systolic blood pressure [10], onset season [11], anemia, cognitive function [12], and whether intervention surgery was performed [13]. However, conflicting evidence exists regarding the relationship between ejection fraction (EF) and readmission within one year. A Swiss study reported that patients with HFmrEF had a higher one-year readmission rate than patients with heart failure with reduced ejection fraction (HFrEF) [14] (34% vs. 30%). In contrast, studies in the United States and Spain found that patients with HFrEF had higher readmission rates than those with HFmrEF [15,16]. Another Spanish study found no statistically significant difference in HF hospitalization between HFmrEF and HFrEF patients [17].

Additionally, patients with HF are typically older and often present with multiple chronic conditions. When these conditions are managed according to clinical guidelines, polypharmacy becomes unavoidable. Polypharmacy increases the risk of adverse drug events, medication errors, and poor adherence [18], all of which can raise the likelihood of rehospitalization, making effective medication management crucial for patients with CHF. Medication literacy serves as a vital link between complex medication regimens and successful patient self-management. Medication literacy is defined as “the degree to which individuals can obtain, comprehend, communicate, calculate, and process patient-specific information about their medications to make informed medication and health decisions to use their medications safely and effectively, regardless of the mode by which the content is delivered (e.g., written, oral, and visual)” [19]. It can act as a key predictor of the rational use of medications [20].

However, research on whether inadequate medication literacy contributes to a higher risk of hospitalization remains limited. Therefore, we conducted this study to investigate one-year readmission in CHF patients and the associated factors, with a particular focus on the relationship between medication literacy and readmission in Chinese CHF patients. Our study may offer novel strategies for healthcare practitioners to identify target patients and key clinical issues that require tailored interventions to reduce readmission rates in patients with chronic diseases.

## Materials and methods

Study population

This retrospective cohort study involved CHF patients diagnosed and treated in three tertiary hospitals in Changsha, Hunan province, China, and was conducted from June 2022 to June 2023. The follow-up period for one-year readmission was extended until June 2024. We obtained data from the electronic medical record database of the hospitals. Participants of the inclusion criteria were aged 18 years and above, had a confirmed CHF diagnosis according to the International Classification of Diseases, 10th Revision (ICD-10) [21], and the New York Heart Association Functional Classifications (NYHA class) [22] was II-IV. Because our study aimed to examine one-year all-cause readmission rates, all patients who died during the index hospitalization were not included. A total of 480 patients were finally enrolled in the study. The study was approved by the Ethics Committee of the Third Xiangya Hospital of Central South University (no: 24430). The requirement for informed consent from participants was omitted due to the retrospective nature of the study.

Data collection

The investigators rigorously selected study participants. Two cardiology nurses, who received standardized training on data collection and quality control, conducted the review of electronic medical records. Data on patients’ demographics, medication literacy, clinical information, laboratory indicators, and all-cause readmission rates were extracted from the hospital’s electronic medical record systems. Medication literacy was assessed using a questionnaire at patient admission and recorded in the medical records. The Chinese version of the Medication Literacy Scale was translated and adapted by Zheng et al. [23]. The scale demonstrated a retest reliability of 0.885, a split-half reliability of 0.840, and a K-R20 coefficient of 0.82 [23].

The scale consists of 14 items, each scored on a 2-point scale (1 for a correct answer and 0 for an error), with higher scores indicating higher patient medication literacy. The maximum score is 14. Based on the calculation formula for discrimination used in educational statistics [24], the scores were classified into three groups: adequate literacy(>10), marginal literacy (4-10, 4 and 10 were included), and inadequate literacy (<4). Thus, higher scores reflect higher levels of medication literacy. To ensure data quality, after one investigator entered the data, a second investigator proofread it to confirm accuracy.

Statistical analysis

Baseline characteristics were compared between patients who experienced all-cause readmission within the one-year follow-up and those who did not. Continuous variables are presented as means ± standard deviations (SD), and categorical variables are reported as numbers and percentages. Comparisons of means were performed using the two independent samples t-test. Non-ordered categorical variables were analyzed using the Chi-squared test or Fisher’s exact test, two-level ordered categorical variables using the Wilcoxon rank-sum test, and multiple-level ordered categorical variables using the Kruskal-Wallis H test.

The rates of all-cause and HF readmission from one month to one year after the index discharge within the one-year follow-up were analyzed. All baseline variables were entered into the univariable analysis model, and those with a *p*-value <0.1 were further included in the multivariable model. To identify factors influencing all-cause readmission within one year, binary logistic regression was performed using the forward selection method. All tests were two-tailed, and *p*<0.05 was considered statistically significant. Statistical analyses were conducted using SPSS Statistics 25.0 software (IBM Corp., Armonk, NY).

## Results

Baseline characteristics of the cohort

Baseline characteristics of the cohort are shown in Table 1. The univariate analysis showed no significant differences in demographic variables between readmitted and non-readmitted patients with CHF. There was a significant difference in medication literacy between the readmitted and non-readmitted groups, with a lower mean medication literacy score in the readmitted group (6.97 vs. 7.99, *p<*0.001). Patients with a longer disease duration had a higher proportion of readmission in one year (6-10 years: 22.9% vs. 21.8%, *p*=0.011). Patients readmitted within one year were more likely to have chronic obstructive pulmonary disease, dilated cardiomyopathy, myocardial infarction, renal dysfunction, and obesity. Differences in HF type were observed; readmitted patients had a lower proportion of HFrEF compared with non-readmitted patients (38.0% vs. 39.8%, *p*=0.039). There was a significance associated with the NYHA Class. In readmitted patients, NT-proBNP was higher than in non-readmitted patients. Diuretics were more frequently used in patients readmitted.

**Table 1:** Baseline characteristics (grouped by whether readmitted for all-cause reasons in one year) (N=480) ^a^Student's t-test was used to compare means; ^b^Chi-squared test or Fisher's exact test was used to compare non-ordered categorical variables; ^c^Wilcoxon rank-sum test was used to compare two ordered categorical variables; ^d^Kruskal-Wallis H test was used to compare multiple ordered categorical variables SD: standard deviation; SBP: systolic blood pressure; DBP: diastolic blood pressure; COPD: chronic obstructive pulmonary disease; HFrEF: heart failure with reduced ejection fraction; HFmrEF: heart failure with mid-range ejection fraction; HFpEF: heart failure with preserved ejection fraction; NYHA: New York Heart Association; IQR: interquartile range; ACEI: angiotensin-converting enzyme inhibitor; ARB: angiotensin receptor blocker; SGLT-2 inhibitors: sodium-glucose cotransporter-2 inhibitors

Variables	Overall (n=480)	Readmitted (n=279)	Not readmitted (n=201)	*P*-value
Demographics				
Age, years, mean (SD)	65.9 (12.7)	66.2 (13.2)	65.5 (11.7)	0.607^a^
Female, n (%)	158 (32.9)	99 (35.5)	59 (29.3)	0.785^b^
SBP, mmHg, mean (SD)	122.4 (21.1)	121.1 (21.8)	124.2 (20.0)	0.168^a^
DBP, mmHg, mean (SD)	75.4 (13.4)	73.9 (13.0)	77.6 (13.7)	0.141^a^
Heart rate, mean (SD)	77.0 (13.1)	77.3 (14.1)	76.6 (11.5)	0.602^a^
Medication literacy, mean (SD)	7.48 (2.45)	6.97 (2.23)	7.99 (2.56)	<0.001^a^
Disease duration, years, n (%)				0.011^d^
<1	166 (34.6)	84 (30.1)	82 (40.8)	
1-3	120 (25.0)	65 (23.4)	55 (27.4)	
4-5	86 (17.9)	51 (18.3)	34 (16.9)	
6-10	108 (22.5)	64 (22.9)	44 (21.8)	
Comorbidities, n (%)				
Dilated cardiomyopathy	84 (17.5)	50 (17.9)	34 (16.9)	0.020^b^
Hypertension	37 (7.7)	27 (9.7)	10 (5.0)	0.829^b^
Vascular disease	24 (5.0)	13 (4.7)	11 (5.5)	0.214^b^
Atrial fibrillation	36 (7.5)	10 (3.6)	26 (12.9)	0.093^b^
Diabetes	168 (35.0)	109 (39.1)	59 (29.4)	0.057^b^
Myocardial infarction	111 (23.1)	78 (27.9)	33 (16.4)	0.013^b^
Chronic kidney disease	280 (58.3)	143 (51.3)	137 (68.2)	0.001^b^
COPD	87 (25.7)	50 (25.4)	37 (18.4)	0.010^b^
Obesity	57 (11.9)	31 (11.1)	26 (12.9)	0.030^b^
HF type, n (%)				0.039^d^
HFrEF (EF <40%)	186 (38.8)	106 (38.0)	80 (39.8)	
HFmrEF (EF 40-49%)	126 (26.2)	85 (30.5)	41 (20.4)	
HFpEF (EF ≥50%)	168 (35.0)	88 (31.5)	80 (39.8)	
NYHA class, n (%)				<0.001^d^
II	104 (21.7)	42 (15.1)	62 (30.8)	
III	196 (40.8)	126 (45.2)	70 (34.8)	
IV	180 (37.5)	108 (38.7)	72 (35.8)	
Laboratory indices, median (IQR)				
NT-proBNP, pg/ml	3825 (1456, 8401)	4161 (2021, 7961)	3448 (1514, 6881)	0.045^c^
Haemoglobin, g/L	122 (105, 132)	121 (104, 132)	123 (103, 129)	0.675^c^
Creatinine, μmol/L	105 (82, 151)	112 (86, 158)	107 (82, 155)	0.040^c^
Sodium, mmol/L	138 (136, 146)	139 (137, 146)	138 (135, 141)	0.758^c^
Medications at discharge, n (%)				
Beta-blockers	423 (88.1)	249 (89.2)	174 (86.6)	0.313^b^
ACEIs/ARBs	365 (76.0)	203 (72.8)	162 (80.6)	0.087^b^
SGLT-2 inhibitors	223 (46.5)	133 (47.7)	90 (44.8)	0.087^b^
Mineral corticoid antagonist	60 (12.5)	42 (15.1)	18 (9.0)	0.154^b^
Loop diuretics	383 (79.8)	232 (83.2)	151 (75.1)	0.046^b^

Burden of all-cause or heart failure readmission during one-year follow-up in patients with chronic heart failure

**Figure 1:**
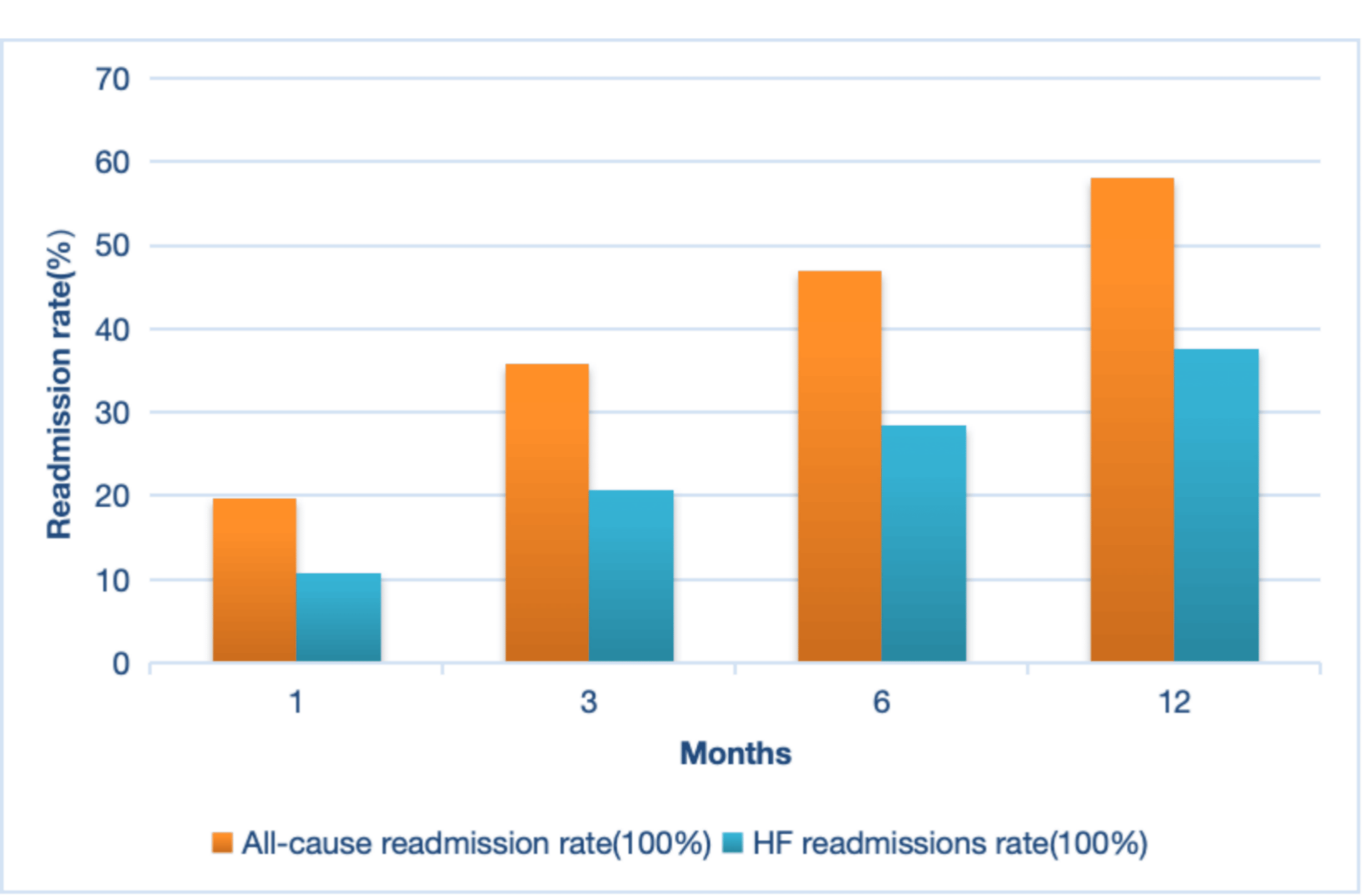
Prevalence of all-cause or HF readmissions at different times after discharge within one year HF: heart failure

The study included a total of 480 patients with CHF. The results showed that the all-cause readmission rates at one month, three months, six months, and one year were 19.7%, 35.8%, 46.9%, and 58.1%, respectively, while the corresponding proportions for HF readmissions were 10.8%, 20.7%, 28.4%, and 37.6% (Figure 1).

Association between possible factors and readmission within one year in patients with chronic heart failure

**Figure 2:**
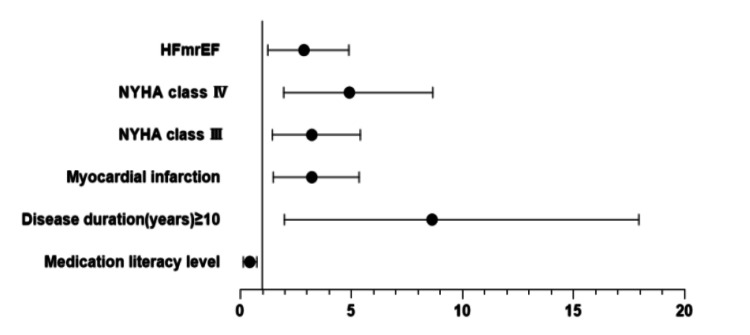
Multivariate analysis of factors associated with all-cause readmission within one year in CHF patients (N=480) NYHA: New York Heart Association; HFmrEF: heart failure with mid-range ejection fraction

Table 2 and Figure 2 show the result of binary logistic regression using the all-cause readmission within one year as the dependent variable and all variables with *p*<0.10 in the univariate analysis as the independent variables. The results identified the following risk factors of CHF readmission: disease duration ≥10 years (OR=5.961, 95% CI=1.981-17.939, *p*=0.001), myocardial infarction (OR=2.818, 95% CI=1.486-5.344, *p*=0.002), NYHA class Ⅲ (OR=2.794, 95% CI=1.442-5.411, *p*=0.002), NYHA class Ⅳ (OR=4.112, 95% CI=1.952-8.664, p<0.001), and HFmrEF (OR=2.463, 95% CI=1.241-4.889, *p*=0.010), while higher medication literacy (OR=0.411, CI=0.134-0.743, *p*=0.013) was a protective factor. Patients with higher medication literacy levels were less likely to be readmitted.

**Table 2:** Multivariate analysis of factors associated with all-cause readmission within one year in CHF patients (N=480) CHF: chronic heart failure; OR: odds ratio; CI: confidence interval; NYHA: New York Heart Association; HFmrEF: heart failure with mid-range ejection fraction

Variables	B	P	OR	95% CI	
				Lower	Upper
Medication literacy level	-1.142	0.013	0.411	0.134	0.743
Disease duration ≥10 years	1.785	0.001	5.961	1.981	17.939
Myocardial infarction	1.036	0.002	2.818	1.486	5.344
NYHA class					
III	1.027	0.002	2.794	1.442	5.411
IV	1.414	<0.001	4.112	1.952	8.664
HFmrEF	0.901	0.010	2.463	1.241	4.889

Specifically, patients with disease duration ≥10 years had a 5.961 times higher risk of readmission within one year than those with disease duration <1 year. Patients with a previous myocardial infarction had a 2.818 times higher risk of being readmitted within one year than patients without a myocardial infarction. The risk of readmission within one year was 2.794 and 4.112 times higher in patients with NYHA class III and IV than in patients with NYHA class II. The risk of readmission within one year was 2.463 times higher in patients with HFmrEF than in patients with HFrEF.

## Discussion

Among this cohort of HF patients, early post-discharge hospital readmissions occurred frequently, predominantly driven by worsening HF. In our study, we investigated the one-year readmission rate in Chinese CHF patients and explored the predictors of readmission. Our results showed that 58.1% of CHF patients were readmitted to the hospital within one year, which was higher than the 31% reported by Hu et al. [8] in Jiangsu, China, and the 38.5% reported by Chang et al. [25] in Taiwan. This difference may be explained by variations in the study samples. Hu et al.’s study only included patients diagnosed with HFrEF, while our study included all HF types based on ejection fraction (HFrEF, HFmrEF, and HFpEF).

As evidenced in our study, patients with HFmrEF were more likely to be readmitted within one year, which may explain the relatively higher readmission rate in our study compared with Hu et al.’s study [8]. Chang et al.’s study [25] included patients with acute heart failure, while our study included patients with CHF, who are more likely to be readmitted within one year due to the chronic and recurrent nature of the disease. The high readmission rate of CHF highlights the need for increased research focus and clinical efforts to prevent readmission. Our study also showed that the mean medication literacy score of readmitted CHF patients was lower (6.97 vs. 7.99), indicating that the medication literacy among CHF patients still has room for improvement.

Our study showed that medication literacy is an important factor affecting readmission within one year in patients with chronic heart failure, according to logistic regression analysis. The possible reason is that improving medication literacy can prevent a significant proportion of medication-related adverse events, thereby reducing readmissions among heart failure patients caused by inappropriate medication use [26]. A clear understanding of medication literacy is crucial for developing effective strategies to ensure proper medication use, which directly impacts clinical outcomes [27].

Low medication literacy represents an important, modifiable, non-clinical risk factor for readmission in patients with chronic heart failure. Therefore, integrating the improvement of medication literacy into the standard management pathway for heart failure and implementing multidisciplinary, tailored, and sustained interventions can effectively enhance patients’ self-efficacy, optimize treatment outcomes, and serve as an essential approach to reducing healthcare burdens while achieving patient-centered chronic disease management. Future research and clinical practice should place greater emphasis on developing scalable, culturally adapted intervention models and conducting long-term evaluations of their cost-effectiveness.

Besides medication literacy, HFmrEF, disease duration ≥10 years, NYHA class Ⅲ, NYHA class Ⅳ, and myocardial infarction were shown by logistic regression to be significant determinants of readmission. Therefore, an integrated, multifaceted approach is needed to reduce the one-year readmission rate in CHF patients.

The study identified HFmrEF as a risk factor for one-year readmission among CHF patients. The risk of readmission within one year was 2.463 times higher in HFmrEF patients than in HFrEF patients. This finding was consistent with the results of Cheng et al. [28]. One major reason may be that patients with HFmrEF are more susceptible to infections, which predispose them to worsening heart disease [29]. Infection is a major trigger for the onset of CHF [30], exacerbating symptoms and increasing the risk of rehospitalization in CHF patients. It is suggested that healthcare professionals strengthen infection prevention measures for HFmrEF patients during hospitalization. In addition, patients and their families should receive education and training on infection prevention before discharge. Regular vaccinations after discharge are also recommended to reduce the risk of infections and prevent readmission [31].

Another risk factor for readmission was longer disease duration. The study showed that CHF patients with disease duration ≥10 years had a 5.961 times higher risk of readmission than patients with disease duration <1 year. This finding was consistent with the study by Butt et al. [32], who found that CHF patients with longer disease duration had a higher readmission rate than patients with new-onset HF, and identified disease duration as a significant risk factor for readmission. Multiple factors may explain the positive association between disease duration and readmission risk, including increasing age [33], malnutrition [34], and poor medication adherence as the disease progresses, which may lead to continuous deterioration of cardiac function and thus increase the risk of readmission. It is therefore suggested that more attention be paid to CHF patients with longer disease duration, and that effective preventive measures be implemented to mitigate all potential risk factors that may lead to readmission.

The study also showed that poor heart function was a risk factor for readmission within one year. Compared to CHF patients with NYHA class II, the risk of readmission among patients with NYHA class III and IV increased by 2.794 and 4.112 times, respectively. This finding was consistent with most previous studies. For instance, Niu et al. conducted a study among 1,727 Chinese CHF patients and found that heart function was a risk factor for hospital readmission [35]. Another study also showed that NYHA class IV was a major influencing factor for readmission in CHF patients [36]. This may be related to the deterioration of cardiac structure and function, leading to reduced cardiac tolerance in CHF patients. The decreased cardiac load tolerance can result in symptoms such as chest tightness and fatigue in daily life [37], which further increases the risk of hospital readmission. It is recommended that CHF patients initiate medication or symptomatic therapy as early as possible to maintain cardiac function. For patients with poorer cardiac function, interventional procedures such as pacemakers or heart transplantation can be considered. Given the high costs associated with interventional heart surgery, early prevention and management of cardiac function is strongly advised.

Consistent with previous studies, the study showed that a previous myocardial infarction is a risk factor for readmission. CHF patients who had previously experienced myocardial infarction had a 2.818 times higher risk of readmission within one year compared to patients without myocardial infarction. One study showed that 12-13% of CHF patients with previous myocardial infarction had a poor prognosis after hospital discharge [38]. Another study in the United States showed that CHF patients with myocardial infarction were more likely to require hospital treatment again after discharge [39]. This may be because patients with previous myocardial infarction have poorer cardiac function and reduced cardiac compensatory ability, which may be insufficient to meet the demands of daily life or exercise, leading to symptoms such as fatigue and chest tightness and resulting in readmission [40]. Percutaneous coronary intervention can be undertaken in patients who have had a myocardial infarction, and the reduction in readmission following myocardial infarction treatment has been well documented [41].

The major strength of this study is that we identified medication literacy as being related to all-cause readmission in patients with chronic heart failure, whereas most previous studies focused on clinical and laboratory parameters. The results of this study highlight the impact of medication literacy on readmission. This study also has several limitations. First, the subjects were recruited from three tertiary hospitals in Hunan, so the results may not be generalizable to all of China. Second, this was a retrospective study, and the associated limitations should be considered. Third, the study size was relatively small, and improvements are needed in handling missing values and statistical analysis methods. In the future, longitudinal surveys across multiple regions, populations, and extended time periods could be conducted to more deeply explore the factors influencing readmission in CHF patients.

## Conclusions

Our study found that the risk factors for all-cause readmission within one year among CHF patients included lower medication literacy, in addition to HFmrEF, disease duration ≥10 years, previous myocardial infarction, NYHA class III, and NYHA class IV. Our research provides both practical and theoretical insights to guide decision-makers and inform future research agendas. These findings offer valuable guidance for the development of prevention and intervention programs aimed at improving the prognosis and overall well-being of CHF patients.

## References

[1] (2018). Global, regional, and national incidence, prevalence, and years lived with disability for 354 diseases and injuries for 195 countries and territories, 1990-2017: a systematic analysis for the Global Burden of Disease Study 2017. Lancet.

[2] (2022). Report on cardiovascular health and diseases in China 2021: an updated summary. Biomed Environ Sci.

[3] Groenewegen A, Rutten FH, Mosterd A, Hoes AW (2020). Epidemiology of heart failure. Eur J Heart Fail.

[4] Savarese G, Lund LH (2017). Global public health burden of heart failure. Card Fail Rev.

[5] Tay WT, Teng TK, Simon O (2021). Readmissions, death and its associated predictors in heart failure with preserved versus reduced ejection fraction. J Am Heart Assoc.

[6] Jencks SF, Williams MV, Coleman EA (2009). Rehospitalizations among patients in the Medicare fee-for-service program. N Engl J Med.

[7] Duflos C, Troude P, Strainchamps D, Ségouin C, Logeart D, Mercier G (2020). Hospitalization for acute heart failure: the in-hospital care pathway predicts one-year readmission. Sci Rep.

[8] Hu Y, Wang X, Xiao S (2022). Development and validation of a nomogram model for predicting the risk of readmission in patients with heart failure with reduced ejection fraction within 1 year. Cardiovasc Ther.

[9] Omersa D, Farkas J, Erzen I, Lainscak M (2016). National trends in heart failure hospitalization rates in Slovenia 2004-2012. Eur J Heart Fail.

[10] Huang X, Liu J, Zhang L (2022). Systolic blood pressure and 1-year clinical outcomes in patients hospitalized for heart failure. Front Cardiovasc Med.

[11] Wang N, Farrell M, Hales S, Hanvey K, Robertson G, Sharp P, Tofler G (2020). Prevalence and seasonal variation of precipitants of heart failure hospitalization and risk of readmission. Int J Cardiol.

[12] Son YJ, Jang I (2023). One-year trajectories of self-care behaviours and unplanned hospital readmissions among patients with heart failure: a prospective longitudinal study. J Clin Nurs.

[13] Wang F, Sterling LH, Liu A, Brophy JM, Paradis G, Marelli A (2020). Risk of readmission after heart failure hospitalization in older adults with congenital heart disease. Int J Cardiol.

[14] Rickenbacher P, Kaufmann BA, Maeder MT (2017). Heart failure with mid-range ejection fraction: a distinct clinical entity? Insights from the Trial of Intensified versus standard Medical therapy in Elderly patients with Congestive Heart Failure (TIME-CHF). Eur J Heart Fail.

[15] González-Guerrero JL, Paredes-Galán E, Ferrero-Martínez AI (2020). Characteristics and one-year outcomes in elderly patients hospitalised with heart failure and preserved, mid-range and reduced ejection fraction (Article in Spanish). Rev Esp Geriatr Gerontol.

[16] Shah KS, Xu H, Matsouaka RA (2017). Heart failure with preserved, borderline, and reduced ejection fraction: 5-year outcomes. J Am Coll Cardiol.

[17] Farré N, Lupon J, Roig E (2017). Clinical characteristics, one-year change in ejection fraction and long-term outcomes in patients with heart failure with mid-range ejection fraction: a multicentre prospective observational study in Catalonia (Spain). BMJ Open.

[18] Beezer J, Clark AL, Todd A, Kingston A, Husband A (2025). The association between polypharmacy and mortality in patients with heart failure: results from the PULSE dataset. ESC Heart Fail.

[19] Raynor DK (2008). Medication literacy is a 2-way street. Mayo Clin Proc.

[20] Pouliot A, Vaillancourt R, Stacey D, Suter P (2018). Defining and identifying concepts of medication literacy: an international perspective. Res Social Adm Pharm.

[21] (2025). International Statistical Classification of Diseases and Related Health Problems (ICD). http://www.who.int/classifications/icd/en/.

[22] The Criteria Committee of the New York Heart Association (1994). Nomenclature and Criteria for Diagnosis of Diseases of the Heart and Great Vessels (9th ed.). https://u1-cd-professional.sc.heart.org/en/guidelines-and-statements/classification.

[23] Zheng F, Zhong Z, Ding S, Luo A, Liu Z (2016). Modification and evaluation of assessment of  medication literacy (Article in Chinese). Zhong Nan Da Xue Xue Bao Yi Xue Ban.

[24] Wei HZ, Zhou RL, Ma JS (1999). Educational Statistics and Measurement.

[25] Chang HY, Wang CC, Wu YW (2017). One-year outcomes of acute decompensated systolic heart failure in Taiwan: lessons from TSOC-HFrEF registry. Acta Cardiol Sin.

[26] Wideqvist M, Cui X, Magnusson C, Schaufelberger M, Fu M (2021). Hospital readmissions of patients with heart failure from real world: timing and associated risk factors. ESC Heart Fail.

[27] Zheng F, Ding S, Lai L, Liu X, Duan Y, Shi S, Zhong Z (2019). Relationship between medication literacy and medication adherence in inpatients with coronary heart disease in Changsha, China. Front Pharmacol.

[28] Cheng RK, Cox M, Neely ML (2014). Outcomes in patients with heart failure with preserved, borderline, and reduced ejection fraction in the Medicare population. Am Heart J.

[29] Farmakis D, Simitsis P, Bistola V (2017). Acute heart failure with mid-range left ventricular ejection fraction: clinical profile, in-hospital management, and short-term outcome. Clin Res Cardiol.

[30] Drozd M, Garland E, Walker AM (2020). Infection-related hospitalization in heart failure with reduced ejection fraction: a prospective observational cohort study. Circ Heart Fail.

[31] Bhatt AS, DeVore AD, Hernandez AF, Mentz RJ (2017). Can vaccinations improve heart failure outcomes?: Contemporary data and future directions. JACC Heart Fail.

[32] Butt JH, Fosbøl EL, Gerds TA (2020). Readmission and death in patients admitted with new-onset versus worsening of chronic heart failure: insights from a nationwide cohort. Eur J Heart Fail.

[33] Bui AL, Horwich TB, Fonarow GC (2011). Epidemiology and risk profile of heart failure. Nat Rev Cardiol.

[34] Liu J, Liu J, Wang J (2022). Prevalence and impact of malnutrition on readmission among hospitalized patients with heart failure in China. ESC Heart Fail.

[35] Clar C, Oseni Z, Flowers N, Keshtkar-Jahromi M, Rees K (2015). Influenza vaccines for preventing cardiovascular disease. Cochrane Database Syst Rev.

[36] Niu XN, Wen H, Sun N, Zhao R, Wang T, Li Y (2022). Exploring risk factors of short-term readmission in heart failure patients: a cohort study. Front Endocrinol (Lausanne).

[37] Al-Tamimi MA, Gillani SW, Abd Alhakam ME, Sam KG (2021). Factors associated with hospital readmission of heart failure patients. Front Pharmacol.

[38] Guazzi M, Wilhelm M, Halle M (2022). Exercise testing in heart failure with preserved ejection fraction: an appraisal through diagnosis, pathophysiology and therapy - a clinical consensus statement of the Heart Failure Association and European Association of Preventive Cardiology of the European Society of Cardiology. Eur J Heart Fail.

[39] Shah RV, Holmes D, Anderson M, Wang TY, Kontos MC, Wiviott SD, Scirica BM (2012). Risk of heart failure complication during hospitalization for acute myocardial infarction in a contemporary population: insights from the National Cardiovascular Data ACTION Registry. Circ Heart Fail.

[40] Nazir S, Minhas AM, Kamat IS (2021). Patient characteristics and outcomes of type 2 myocardial infarction during heart failure hospitalizations in the United States. Am J Med.

[41] Bahit MC, Kochar A, Granger CB (2018). Post-myocardial infarction heart failure. JACC Heart Fail.

